# Development of a hybrid automated chart checking, data collection, and analysis system

**DOI:** 10.1002/acm2.70161

**Published:** 2025-07-14

**Authors:** Edward L. Clouser, Quan Chen, Daniel P. Harrington, Yi Rong, Courtney R. Buckey

**Affiliations:** ^1^ Department of Radiation Oncology Mayo Clinic Arizona Phoenix Arizona USA

**Keywords:** Chart check automation, Chart check database, Plan attributes

## Abstract

**Purpose:**

To describe the creation and clinical deployment of an integrated stand‐alone hybrid automated chart review platform that is comprised of a database, oncology information system (OIS) interfaces, and a user interface (UI).

**Methods:**

A consensus checklist was developed to homogenize the practices of clinical physics staff and physics residents for all plan types. Critical review of the single checklist's items led to the creation of a new concept, the “plan attribute,” which permits specialization of the final checklist based on the characteristics of a particular plan. Only relevant and applicable tests are automatically populated to form a plan checklist based on plan attributes. Each populated checklist in this hybrid automation platform includes elements of full automation, steps or functions that use automation to augment a human input or effort, as well as steps completed by humans. Logging and categorizing of deficiencies found during the chart check was also implemented.

**Results:**

Chart check data was analyzed for an over 4‐year time span starting from the clinical deployment of the tool in October 2020. This analysis included a total of 427 073 tests spread among 7882 initial chart checks. The recorded deficiencies at three triage levels showed 7116 minor errors, 679 major errors, and 71 plan rejections. The plan rejection level is the highest chart deficiency, which required the plan to be rejected and sent back for serious remediation and/or new plan generation.

**Conclusion:**

This report describes the successful development and implementation of a hybrid‐automated chart checking program. The software leverages modern database and software design to improve the per case performance and experience of a multi‐user group. Chart check database permits high‐level review and improvement in all aspects of plan check and user performance.

## 1 INTRODUCTION

In radiation oncology, the radiotherapy (RT) plan quality assurance (QA) or patient chart check is an important safety barrier and has been referenced as the most effective quality control process.^1^, ^2^ Per AAPM Task Group 275 (TG‐275), which conducted a risk‐based analysis of chart checking,^3^ “The review of RT treatment plans and charts by a qualified medical physicist is a key component to ensuring safe, high‐quality care.” Furthermore, Medical Physics Practice Guideline 11a (MPPG‐11a)^4^ helped define a standard for conducting the checks. It included multiple tables with recommendations while also indicating that these checks go beyond work done only by physicists.

TG‐275 (table S1.A.III) indicates that a plan check should include tests pertaining to patient assessment and intent, simulation, and treatment planning. Within the treatment planning category, the report gives examples that include tests of the contouring, prescription, adherence to a standard operating procedure (SOP), dose distribution, dose verification, isocenter placement, image guidance and other ancillary systems, scheduling, and others. Despite over 75 items included in the checklist, it is still not a comprehensive list, as there may be additional institution, hardware/software device, or even workflow specific tests. Additionally, failure mode and effects analysis (FMEA) is utilized for designing tests or tasks to mitigate potential risks not considered or prioritized in TG‐275.^5^

Many other factors play a role in conducting a quality plan and chart review, and these are also covered in recent literature.^6^ Multiple physicist practices must account for variability in staff training, availability, speed, distraction/fatigue, and thoroughness, as well as the likely inconsistent pace of new work. The use of checklists as a safety barrier has been widely adopted in clinics and elaborated in literature.^7^, ^8^ Within the medical physics community at large, the value of automation is recognized, but the robustness of automation alone is often questioned.^9^, ^10^ Automated chart checking has been proposed and successfully attempted at several institutions with software implementations specific to their needs.^11^, ^12^, ^13^, ^14^, ^15^, ^16^, ^17^, ^18^ There are examples prevalent to specific problems within the chart checking realm where automation could play a direct role.^19^, ^20^ Nevertheless, there is currently not an all‐inclusive vendor solution, most likely due to the highly customized design needs based on individual institutions’ workflows.

This work describes a hybrid automated chart checking tool that was implemented and accommodates a relatively large‐scale radiation oncology operation, locally referred to as “Chartist.” The design and implementation of the user experience (UX) are described to assist the community in developing a generalizable solution. The software utility described here serves multiple groups (sections within the software) within the department and can support checklists comprising tests that are checked fully manually, fully automated, or in a hybrid fashion. This work focuses solely on the physics initial chart check workflow for the photon clinic. The architecture and design of Chartist are in the context of a multi‐user, multi‐section environment. The term hybrid automation is used in two contexts: the program uses manual and automated steps, and individual automations may be partially automated, leaving a manual process to complete, also considered hybrid automation.

## 2 METHODS AND MATERIALS

### 2.1 Overall design for Chartist

#### 2.1.1 Checklist development and compartmentation

A consensus checklist was developed for initial chart checking, encompassing all the tests required to validate every plan type for different RT treatments. This master checklist was initially created based on local clinical workflows. Each medical physicist in the group was queried for any additional tests needed for all plan types. The conglomerated list was distributed back to the physics staff, and each item was scrutinized to determine its appropriateness. The master list predated TG‐275 and MPPG‐4a but was vetted against them and has been further expanded and customized over time.

The master list was reviewed by the physics group with a focus on embracing the goal of “explicit, concise, and unambiguous behavior” as described in the guideline. To that end, the endeavor was to keep any chart check's list as short as possible without compromising safety and to prevent user fatigue by populating tests applicable only to specific plan types (e.g., electron, volumetric modulated arc therapy, VMAT), special procedures (e.g., stereotactic radiosurgery, SRS; stereotactic body radiotherapy, SBRT), particular treatment methods (e.g., total skin electron therapy, TSET; total body irradiation, TBI), specific ancillary techniques (e.g., various motion management techniques, various imaging guidance techniques), or certain patient characteristics (e.g., use of bolus). To achieve that, a list of “plan attributes” was created, as shown in Table 1. Every test in the master list was then categorized to indicate when the test was needed. Tests that were needed for all chart checks were placed in the attribute of “General”. The checklist becomes a two‐part process, first identifying the attributes of the plan, then proceeding with checking items that pertain to those attributes and any general tests. Most plans contain several attributes, and no plan contains all of them.

**TABLE 1:** Initial Chart Check plan attributes.

Stereotactic radiosurgery	Total skin electron therapy	Has Shift
Intensity modulated RT	Stereotactic body RT	Multiple dose levels
Multiple isocenters	Gating	Deep inspiration breath hold
4DCT taken	Bolus used	Electronic implanted device
Image guided RT ordered	Previous RT	Image fusion ordered
Patient on clinical protocol	General	BID
Vision RT	ExacTrac	Couch model used
Electron	Triggered imaging	Brass grid
VMAT grid		

Abbreviations: 4DCT, four‐dimensional computed tomography; BID, bis in die (twice daily); RT, radiation therapy; VMAT, volumetric modulated arc therapy.

After compartmentation of the master checklist into smaller checklists associated with various plan attributes, these secondary lists were arranged based on the portion of the oncology information system (OIS) the information being tested resides in. Locally, these categories include ARIA's (the OIS from Varian Medical Systems, Inc., Palo Alto, CA, USA) treatment planning, treatment preparation, documents, care path, plan schedule, reference points, journal note, and prescription, all of which are organized as a tab control in Chartist. In addition, there is a tab labeled “Extramural” for tests that require the user to leave the ARIA environment. More specifically, plan quality related tests would be listed under the “Treatment Planning” tab, while all document related checks would be on the “Documents” tab. Each tab also indicates if the checklist contained within it is complete or not with a checkmark or an “X” graphic.

#### 2.1.2 Test versus task

The sub‐checklist under each tab is further grouped into two types: tests or tasks. A test indicates the user is checking someone else's work and needs to render a response to whether it was done correctly or not. A task indicates the user needs to take an action (e.g., import a file, create a document), and it is there to remind the chart checker to do it; it has a binary state of done or undone. The distinction plays a role when the chart check is completed, as task completion is double‐checked via automation.

#### 2.1.3 Minor, major, and reject

Each test in the checklist has a checkbox to indicate the check has been successfully completed and the level of clinical impact if a deficiency is identified. The software features a triage system, where physicists indicate if their finding was “minor” or “major,” and once remedied, the completion box can be checked, or they could indicate the plan was unsuitable for treatment by selecting the “reject” box. Any system for indicating the severity of a caught error could be used in a general solution for future analysis of recurring errors.

### 2.2 Automations

The local chart check process strictly follows the Care Path functions in ARIA; a task is created in the OIS that indicates the work needs to be done, it becomes active as soon as a series of prerequisite steps are completed, and the task is assigned to a qualified expert to complete, other OIS have similar functionality.^21^ Full automations are employed when the underlying principles have a well‐defined logic such that the algorithm can declare the check successfully completed (done) or detect a discrepancy (minor or major). Partial or hybrid automations can process collected information and present it in a note attached to the test. The automations are directly integrated into the components previously discussed and can be categorized as belonging to one of four main areas: workflow, plan attribute, plan checks, and audit.

#### 2.2.1 Workflow automations

Examples of workflow automations include detecting that a plan is available for checking, importing the data to be checked, or finding and displaying the patient's first treatment appointment. The automated collection, logging, and display of key time stamps can be used for workflow analysis. Some timestamps are directly extracted from the OIS database, while others must be generated by recognizing when a series of predecessor steps are all completed, and still others are related to work completed inside of the chart checking software itself. Chartist also allows for the chart check workload management by displaying the chart checker list imported directly from the physics rotation calendar on Outlook (Microsoft Corporation, Redmond, WA, USA).

#### 2.2.2 Plan attribute automations

Plan attribute automations are used to establish which tests and tasks should be assigned to a specific chart. Most of these automations rely on strict department nomenclature rules and other standardizations. As of this writing, all but two of the plan attributes are automated. The two manual plan attributes include “Patient on Clinical Protocol,” meaning a patient is enrolled in a clinical trial, and “Multiple Isocenters,” meaning multiple plans using different isocenters in one single prescription, such as a craniospinal irradiation.

#### 2.2.3 Plan check automations

Once the relevant tests and tasks are established by the plan attribute automations (and that are manually selected), many of them are evaluated on a partially or fully automated basis through a series of automated plan checks. Full automations are employed when the underlying principles have a well‐defined logic such that the algorithm can declare the check successfully completed or detect a discrepancy.

Partial automations for plan checks are useful when a test has many failure states, but not every failure state can be detected automatically. A test will report a minor or major finding if any automations within that test fail; however, it will not be marked as done even if they all pass, because the user must consider additional (non‐automatable) information before fully evaluating the test. One scenario for the inclusion of hybrid automations is when information is contained within the treatment planning system (TPS) but perhaps hard or time‐consuming to obtain. Hybrid automations pull out and display the data for chart checkers, allowing a streamlined evaluation of the information.

#### 2.2.4 Audit automations

The audit automation occurs after the chart check has been completed for both tests and tasks. For example, the chart checking physicist is required to upload a Monitor Unit second check document for each plan, and upon doing that they can mark that task as done. The audit automation for this task checks if the task is marked as complete even if the document is not uploaded. The audit automation prevents the false manual marking of completion for chart check tasks.

### 2.3 Data sources

The Chartist accesses detailed information from an OIS and TPS to ensure the most accurate data for chart checking. To successfully validate information from both sources, it was required to develop multiple methods for accessing data and implementing automations. A Windows (Microsoft Corporation, Redmond, WA, USA) service was established on a server, which runs every 2 min, facilitating the automations and other needed data exchange between the ARIA database (which also includes the TPS information) and Chartist's database (referred to as the Chartist data service).

An ADO.NET Entity data model of the ARIA database in C# was created, as described by Dr. Rex Cardan on his YouTube channel.^22^ This technique can likely be performed on other databases as well, but this work will only mention ARIA. The database is then accessed via Language Integrated Query (LINQ) in C#. The database includes information beyond the treatment plan, such as journal notes and appointments. The Chartist data service runs on a server that does not have Eclipse TPS installed. Because of this, a separate program to utilize the Eclipse Scripting Application Programming Interface (ESAPI) calls was created, locally referred to as the “ESAPI helper.” The data service and helper execute code and write results to a text file that is ingested by the data service in a later step.

Based on the vendor design, only certain header information and the preview text are stored within the ARIA database, instead of full patient information or documents. For these more nuanced evaluations, Varian's web service Application Programming Interface (API), commonly referred to as the “Gateway,” a standard File Transfer Protocol is used to gather complete documents. For example, the information contained within the physician's simulation order originates from the hospital's electronic medical record, EPIC (Epic Systems Corporation, Verona, WI, USA), and it is translated into ARIA as a structured text document via an interface. All patients will have a simulation order with the same name and highly similar header information and preview text, which can be extracted into the database for test automation.

### 2.4 General software development architecture

**FIGURE 1:**
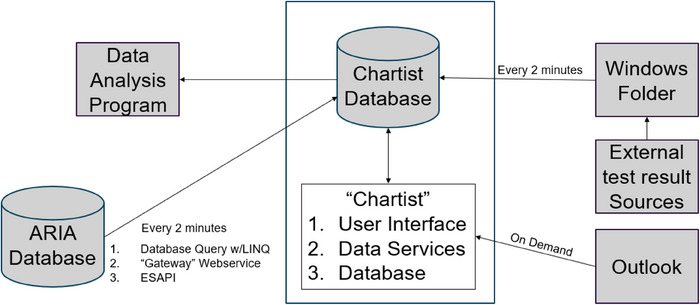
Architecture diagram of the clinical components of Chartist The Chartist program is made of three distinct clinical components, the user interface, a Windows service which automates the collection of data with ARIA and other external data sources, and a dedicated database for storing the results. The database is also accessible by a data analysis program with a separate user interface.

Chartist was developed with the .NET software development framework in the C# programming language. A broad‐level visualization of the architecture is displayed in Figure 1. Within the software, a structured query language (SQL) database hosted on an institutional server was used to facilitate data storage of the chart check functionality. Microsoft Entity Framework in support of LINQ queries was employed to translate data in ARIA's SQL database to the C# programming language to execute automation code.

The graphical user interface (GUI) was customized via an integrated development environment and framework (Windows Presentation Foundation—Microsoft Corporation) employing Extensible Application Markup Language (XAML), a declarative language based on XML. In general, XAML was utilized to define the visual appearance of the application, while code‐behind logic (code associated with markup) was deployed to implement functionality in response to user interactions.

All classes and automation methods are in a separate project, built as the “ChartistAPI.dll,” which allows it to be referenced by Chartist, Chartist data service, and multiple other projects in the department.^23^ An additional user interface was purpose‐built for mining the chart check results and other aggregate data about chart checking to be used for practice improvement, workforce management, and training or education purposes. This arrangement is diagrammed in Figure 1.

In keeping with best practices and institutional requirements for software development, all Chartist code is held in the department's code repository (Azure Development Ops Git) for version control as well as other suggestions made for the development of custom clinical software.^24^

### 2.5 Chartists testing prior to clinical deployment

A separate user interface (UI) was created for testing all automations, in isolation, before they are added to the ChartistAPI.dll and deployed to the clinic. Additionally, testing was conducted by a group of users who are not part of the code development within the department. Each automation was tested for every combination of logic choices it contains. Some automations may contain as many as eight conditions that affect the result, and each of those conditions was tested via a corresponding plan. Additional tests were conducted for the simulated missing information scenario to ensure the code has adequate catches with adequate user alerts. A separate test Chartist database was built for initial code validation and subsequent version upgrade validation to ensure safe clinical rollout. The local rules for Chartist code validation are available in Supporting Information.

Code review was conducted as available but limited by the group membership's knowledge in programming. All the automation code was catalogued on the department's internal web pages with a description of what the code should do in layman's terms, the known limitations, future improvements, and an image of the code itself included for each automation method.

To safeguard the integrity and security of the clinical database, Chartist is not allowed to write‐back to the Eclipse/ARIA environment. The Chartist UI, ChartistAPI.dll, Windows service, testing environment software, ESAPI helper, and data extraction programs are all independent projects with their own version control and numbering.

## 3 RESULTS

**FIGURE 2:**
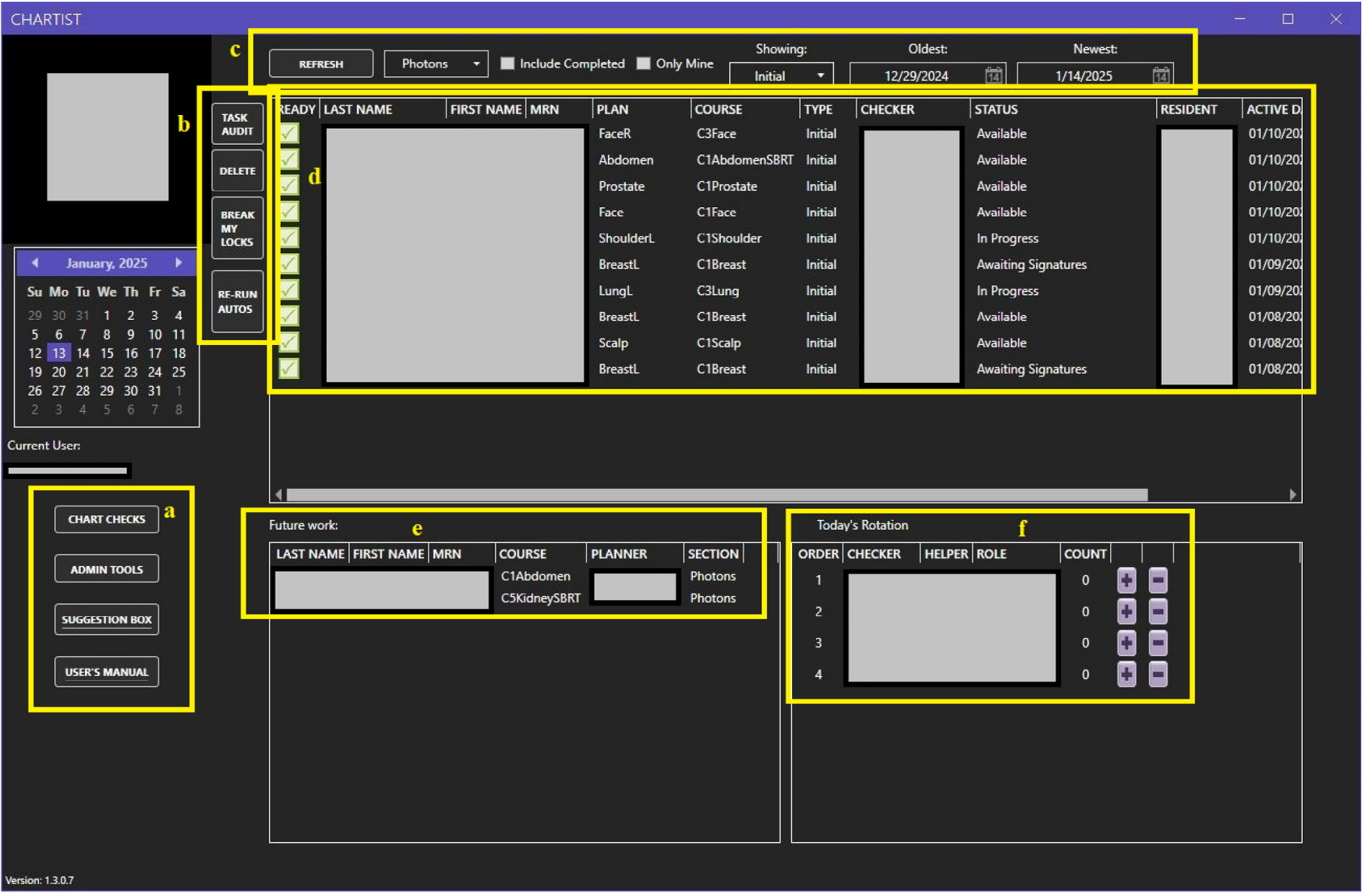
Homepage, where work is selected and dispatched (a) navigation buttons that appear on all screens, including hyperlinks to the suggestion box and user's manual on our departmental SharePoint (b) check utility/management buttons (c) worklist parameters and filters (d) details of individual plans and their current status, as determined by the selections in (c) (e) future work, a list of plans that have received physician approval and are in the final documentation step before being ready for chart check (f) rotation and assignment management tool.

**FIGURE 3:**
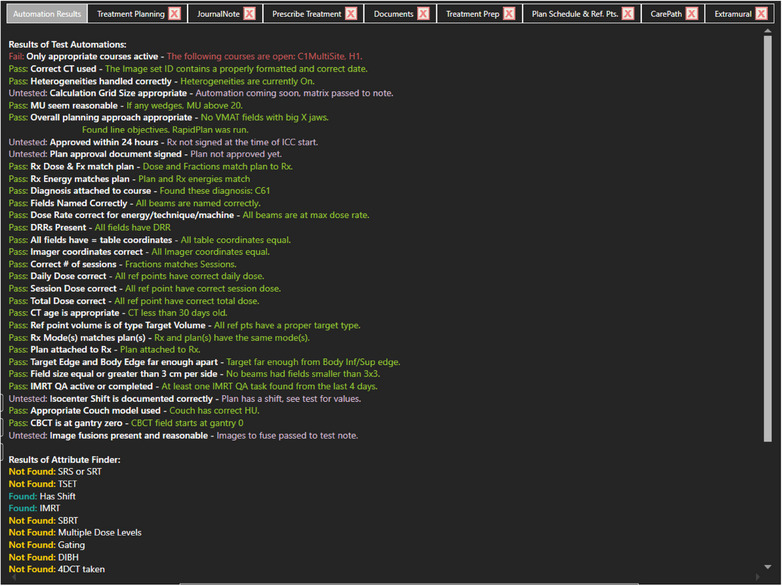
Initial Chart Check, the automation summary report, the user is provided the results and a narrative for any attribute that was found or test that was conducted via automation. If an automation was skipped or it represents some hybrid automations, the user is told via the “Untested” status, and the narrative includes further information.

**FIGURE 4:**
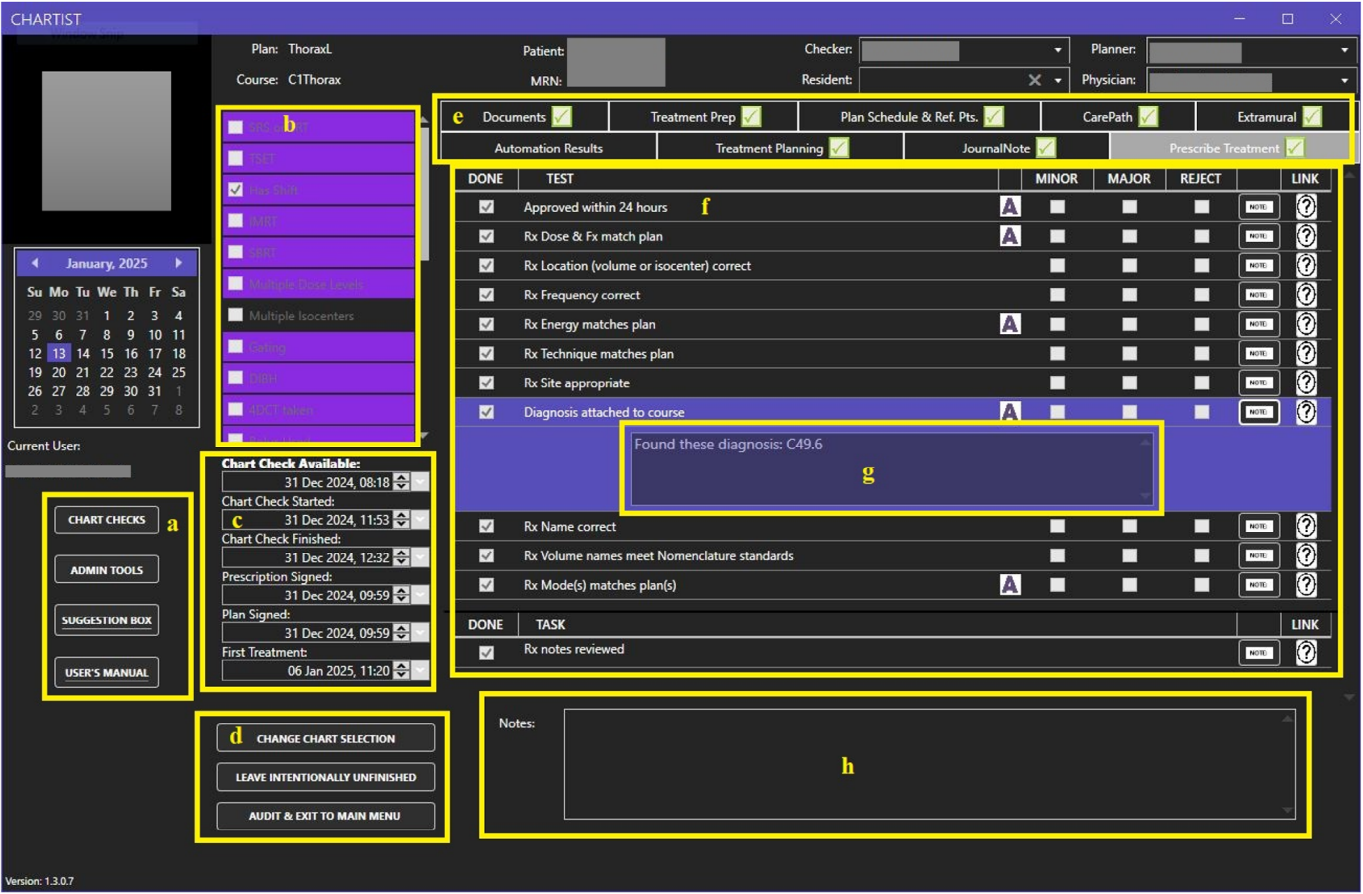
Initial Chart Check Example Checklist (a) Navigation buttons that appear on all screens, including hyperlinks to the suggestion box and user's manual on our departmental SharePoint (b) Full list of our identified plan attributes. Attributes that can be identified by automation are highlighted in purple; those that require manual identification are in black. Any attribute found in the plan will be indicated with a check mark. (c) Key date and time values (d) View specific navigation buttons. The three in this screenshot indicate the three ways to exit an individual plan check and return to the home screen. (e) Tabs to navigate between checklists, with an indicator graphic to indicate if that specific checklist is complete. (f) Test and task checklists specific to that tab. The white and purple “A” icon indicates to a user that some amount of automation was employed for the test. Additional information about any individual test may be found via hover text and/or external links. (g) A note may be left per item utilizing the “Note” button to hide or expose the note space. (h) This space is for notes that relate to the entire check as opposed to an individual test and is visible throughout the chart check regardless of which checklist is currently on the screen.

The software's home screen and chart‐checking interface underwent multiple revisions for both aesthetic and functional content, and the current high‐fidelity home screen is shown in Figure 2. The home screen of the Chartist displays the main list of pending charts, upcoming charts, physicists in rotation, calendar view, and other functions. The goals of the successive iterations were to make refinements in the following areas: to ensure functionality on the clinic's standard‐issue desktop and laptop screens, to display all physicist‐desired information in a clear and concise format, and to support the chart‐checking UX by incorporating best practices of the UX and UI design. Chartist was designed to support a group practice where multiple physicists or residents are simultaneously working on individual chart checks in parallel. Figures 2, 3, and 4 show the Chartist UI. Figure 3 shows the automation report for a typical initial chart check, and Figure 4 shows an example of a checklist set within a single thematic tab.

From the initial full clinical deployment of the Chartist tool on 10/1/2020 through the end of the review period 12/31/2024, a total of 427 073 tests across 7882 initial chart checks were pulled from the Chartist database for analysis. Among these tests, 7116 minor, 679 major, and 71 reject level errors were recorded. Of the 7116 minor errors recorded in Chartist, 3476 (48.8%) were detected by automation and another 3640 (51.2%) were manually detected. A key feature of Chartist is the collection of data that can be used to help determine the frequency and severity of errors caught in aggregate. Table 2 shows the quarterly error rates recorded in Chartist over a 4‐year period. Error detection has transitioned from primarily manual catches to parity between manual and automated, and eventually automation taking the lead in finding errors. Table 2 also indicates that the number of errors, as a percentage of total tests, has decreased over time.

**TABLE 2:** Quarterly error rates as found by automation or manually.

				Automation		Manual			Automation		Manual	
		Initial Chart Checks	Tests	Minor	Major	Minor	Major	Reject	Total	% of tests	Total	% of tests
2020	Q4	352	18 957	150	1	249	38	10	151	0.8	297	1.6
2021	Q1	456	25 108	350	15	350	52	3	365	1.5	405	1.6
	Q2	481	26 343	275	19	298	48	6	294	1.1	352	1.3
	Q3	433	22 741	195	8	260	44	4	203	0.9	308	1.4
	Q4	428	22 359	189	8	221	28	2	197	0.9	251	1.1
2022	Q1	450	23 491	157	15	274	31	3	172	0.7	308	1.3
	Q2	446	23 564	121	15	250	30	8	123	0.5	288	1.2
	Q3	439	23 563	162	9	280	6	5	171	0.7	291	1.2
	Q4	469	25 165	228	16	212	27	2	244	1.0	241	1.0
2023	Q1	476	25 439	221	21	173	33	1	242	1.0	207	0.8
	Q2	479	26 104	180	20	142	13	5	200	0.8	160	0.6
	Q3	486	26 636	231	26	159	9	4	257	1.0	172	0.7
	Q4	411	22 560	222	20	164	24	1	242	1.1	189	0.8
2024	Q1	537	29 614	187	16	167	18	3	203	0.7	188	0.6
	Q2	466	25 786	180	12	153	10	1	192	0.7	164	0.6
	Q3	511	28 375	212	17	147	12	6	229	0.8	165	0.6
	Q4	562	31 268	216	17	141	14	7	233	0.8	162	0.5
Overall		7882	42 7073	3476	242	3640	437	71	3718	0.9	4148	1.0

Chartist data provides a tool for workload management and user performance monitoring. As shown in Table 3, total charts checked and error found rates were listed for 10 users in the department, as well as the average duration for checking charts. The data is the time an individual takes to start the work (available to starting) and to complete the work once started (start to finish). Such personnel related data is only being used as an assisting tool for management.

**TABLE 3:** Timing related data for the 10 most frequent chart checkers in Chartist, October 1, 2020, through December 31, 2024.

					Average elapsed work hours (HH:mm)	
Anon User ID	Total Charts Checked	Tests	Noted errors	% of Tests With a noted error	From available to starting	From start to finish
1	1341	72 467	776	1.0%	4:03	2:43
2	1010	54 705	1803	3.3%	3.40	5:15
3	997	53 792	1066	2.0%	1:36	2:30
4	964	51 523	902	1.8%	7:14	4:31
5	775	41 920	1099	2.6%	10:26	4:40
6	645	35 167	338	1.0%	6:44	4:50
7	628	34 275	269	0.8%	6:46	5:05
8	383	21 002	235	1.1%	5:22	7:18
9	377	20 400	618	3.0%	3:33	3:08
10	234	12 814	394	3.1%	3:23	2:51

The top10 most frequently occurring errors are assessed quarterly. The most frequent errors across a recent quarter are shown in Table 4. Quality improvement efforts are made to reduce or eliminate errors from recurring in the future. Several automations in other parts of the workflow have been crafted or additional second checks added to the treatment planner's script because of these quarterly reviews. At the moment, deployment data of the workflow or script changes is not available, so no additional analysis of their impact is available.

**TABLE 4:** Top 10 most frequently found errors quarterly report example.

Error count	Test
73	Only appropriate courses active
31	Fields named correctly
29	Contours handled correctly
27	Fiducial contours burned in DRRs
24	Patient data is in capsule
17	Rx dose and fractionation match plan
13	Rx volume names meet nomenclature standards
11	Reference points meet nomenclature standards
9	Rx name correct
9	Plan ID meets nomenclature standards

## 4 DISCUSSION

This manuscript outlines the development of a hybrid automated chart checking program and its design architecture, database, and analysis tools. The program has increased the perception of consistency, effectiveness, and efficiency of the initial chart check across staff and trainees. Improvements to the institution's chart checking systems have been achieved in multiple ways. First, the plan attribute‐triggered checklists contain only items germane to the plan being checked. This design increases efficiency and reduces user fatigue. Second, the inclusion of automations can shorten the plan check time and permit the allocated time to be used for more qualitative, subtle, and sophisticated review. Third, Chartist database reports can evaluate user performance, identify repeated errors, and rank error frequencies for subsequent FMEA analysis. Finally, refinements to the UI design helped to maximize the effectiveness of the program while reducing the needed input or interaction from the user.

Table 2 shows that, over the time of this review, fewer deficiencies were caught by manual means and approximately the same number were caught by automation. There are a few factors to consider when attempting to draw conclusions from that data. First, the product was under iterative review and improvement during that time, including the addition of new tests, the removal of old tests that no longer served a clinical need, and the transition of some manual tests to automated versions as the clinical practice caught up with the logical needs of the software. Additional scripts and software have been provided to treatment planners, based on information learned from the Chartist data analysis. However, the errors did not completely disappear, as those programs and scripts have their own failure modes, but it is believed that, by design, most of the findings from the automations outside of Charist are being found and corrected before they ever get to Chartist. Other institutions have reported similar results on error rates.^18^

The user performance data as shown in Table 3 can be used to identify user‐related misuse of the tool and improve compliance. A user may find a discrepancy and fix it but forget to record it in the software. The opposite is also true, if an automation generates a false positive, the user is instructed to uncheck the result that the automation had checked, but this may not have been done. One of the data analysis tools created looks for results where the user and the automation had different answers, and those cases are reviewed to further improve the false negative and false positive rates. Understanding and remedying both is an important maintenance step to ensure staff acceptance of the results, as well as improve automation. A sudden uptick in a mismatch may offer clues to a change in SOP where the “right” answer is no longer following the logic as laid out in the program. Specific results of chart checking have little value to other institutions since they likely do not have the same workflows. However, these results can have great value to the institution itself.

The successful development and deployment of the Chartist program relies on departmental efforts on nomenclature standardization and the construction of, and adherence to, SOP on as many plan types as possible. Future versions of the TPS and ESAPI will likely have additional capabilities, and in‐house developed tools will take over more of the manual steps in plan generation that are still error prone. Other automated tests have been introduced within the department to remove human error as often as possible, but it will not be eliminated. The automated tests must be robust enough to withstand unexpected input, and it is believed that the chart check will always require a human partner to help interpret odd results that do not fit into the expectations of the automations.

Table 4 features a quarterly top 10 list and reveals challenges with nomenclature compliance, despite immense efforts in that regard. The specific content of the reports represented by Table 4 is not important, other than to the institution, but is included as an example of how the inherent data collection of Chartist can be used. In‐house scripts and other tools have been developed and deployed, not unlike other institutions.^25^ A worthwhile finding to share is that most of the “top 10” findings would not cause harm or put the patient or staff at serious risk, which is an overall encouragement for safety outcomes. The noted number of mismatches between the prescription dose and fractionation is related to a change in how multiple plans were being assigned to a common prescription that set off a cascade of false positive results before the automation was altered.

Any chart checking tool will require maintenance as technology and department workflows change or grow. Chartist allows tests, tasks, attributes, and sections to be added or retired as needed. The ability to adapt and future‐proof should be evaluated when considering new tools, whether created in‐house or purchased from a vendor. Clinical practices do not remain static, and they are not expected to, but instead are always looking toward the future. All photon physics projects include a review of proposed QA steps in any new workflows, and this review now includes discussing integration with Chartist. End to end tests of new procedures include chart checking to ensure as seamless as possible a transition to the clinic. Since many of the steps rely on previous steps, this testing will occasionally reveal a flaw in logic that was previously not found.

Because many of the components of a chart check test rely on human‐produced data sources, full automation of the entire test is unlikely to be robust. The hybrid automated approach for these tests can still improve throughput but also handle somewhat frequent exceptions. As of this writing, there are over 40 full or hybrid plan check automations that are available for use, which is driven by the plans’ inclusion/exclusion of 24 attributes. Additional automations are being added as needed. Once a new automation is validated, it is deployed for “observation” by adding the method to the ChartistAPI.dll. The Windows service has a test environment mirror that points to a test Chartist database, which is run for one or more days to ensure no errors populate. If this observation period passes with no noted errors or warnings, the updated file is deployed to production. All incoming chart checks after that will include the new automation's results. New tests, tasks, and attributes are handled by administrative tools to directly manage changes within the Chartist U.I., accessible by trained super users.

A main limitation of hybrid automation is that if a plan attribute or an automation logic depends on an upstream manual step in the process, failure can happen due to upstream human error. Future work includes exploring innovative methods to further reduce upstream manual operations or manual tests/tasks and further utilizing the timing information, as described in Section 2.2.a, to improve workflow by identifying bottlenecks or pinch points in the process. There are also plans to explore if any correlations exist between the urgency of a check, the error type, frequency, or the catch rates.

## 5 CONCLUSION

The Chartist program employs modern database and software design to improve the per‐case performance and experience of the user. It also leverages the aggregate data collected across large numbers of charts to assist systematic error detection, workflow improvement, and user performance improvement. This report provides a comprehensive description of the development and deployment of the Chartist program.

## AUTHOR CONTRIBUTIONS

*Conceptualization*: Edward L. Clouser, Jr., Quan Chen, Daniel P. Harrington, Yi Rong, and Courtney R. Buckey. *Data collection/curation*: Edward L. Clouser, Jr. *Formal data analysis*: Edward L. Clouser, Jr. *Investigation*: Edward L. Clouser, Jr. *Methodology*: Edward L. Clouser, Jr. and Quan Chen. *Writing—original draft*: Edward L. Clouser, Jr. *Platform scripting*: Edward L. Clouser, Jr. *Design*: Edward L. Clouser, Jr. and Courtney R. Buckey. *Automation*: Edward L. Clouser, Jr. *Data curation*: Quan Chen and Daniel P. Harrington. *Writing—primary review*: Quan Chen. *Editing*: Quan Chen, Daniel P. Harrington, Yi Rong, and Courtney R. Buckey. *Platform design*: Daniel P. Harrington. *Writing—review*: Daniel P. Harrington, Yi Rong, and Courtney R. Buckey. *Supervision*: Yi Rong and Courtney R. Buckey. *Platform testing*: Courtney R. Buckey.

## ACKNOWLDGMENTS

The authors would like to acknowledge the contributions of Madeline Foster, M.S., Justin Gagneur, M.A., Gary Ezzell, Ph.D., Suzanne Chungbin, M.S., Mirek Fatyga, Ph.D., Greg Penoncello, D.M.P., Shadi Chitsazzadeh, Ph.D. and Xiangsheng Yan, M.S.; in the development of the master list and early adoption of the program, or ongoing support of the development through clinical use.

## CONFLICT OF INTEREST STATEMENT

All authors have no conflicts of interest

## Supporting information



Supporting Information
