# Supplementary material for: Development of a hybrid automated chart checking, data collection, and analysis system
Source: J Appl Clin Med Phys. 2025 Jul 14;26(7):e70161. doi: 10.1002/acm2.70161 (PMC12257347; doi:10.1002/acm2.70161)
Supplement: Supplementary file 1 — Supporting Information [file ACM2-26-e70161-s001.docx]

Chartist code validation and testing documentation:

**Methodology - What and when to validate**

- Validation will be performed on any item in the "Chartist Suggestions or Improvements" list that has a status of "Ready for Testing"
- Validation will be performed on any item listed in the release notes for the next upcoming version
- Validation will be completed on an ad hoc basis for any bug fixes or urgent changes, as determined in consultation with the programming team
- Except in the case of urgent bug fixes or changes, the programming team will allow at least two weeks for the validation team to complete their testing, allow any sequential fixes to be completed and re-tested, for the public facing documentation to be updated, and for the changes to be presented to the end users prior to release​

**Methodology - How to validate and document**

- All relevant code changes will be available in the "integration" version of Chartist, available to all members of the validation team, at the time testing will commence​
- A list of all​ new code (automation, test, feature, design, functionality, etc) will be created from the combination of any item with a status of "Ready for Testing" in the "Chartist Suggestions or Improvements" list AND any items listed in the anticipatory release notes
  - Any item that appears in the "Chartist Suggestions or Improvements" l​ist will have an ISSUE ID, which will be clearly noted on the validation planning worksheet
- ​All testing that can be accomplished using the two test patients (Chartist, Test Plans 09082020 and Chartist, Test Plans Backup 09082020-2) will be completed with test patients
  - Real patients are permitted for use in scenarios where test patients would not have the requisite attributes, but care must be taken to ensure no disruption of clinically relevant data occurs
- For every item on the validation worksheet, a testing method will be identified as to how to validate
  - Simple visual inspection *can*be a meaningful test, and should be noted
  - Validation plans should include testing for both false-negatives and false-positives
  - Validation plans should clearly lay out the steps, so the testing can be recreated later by an independent reviewer if the need arises
    - ​"We will pull in a plan for checking that has an active "Export VisionRT" carepath item, that is not claimed, and see if the audit properly detects this"
    - "We will pull in a plan for checking that has an active "Export VisionRT" carepath item, that is claimed, and see if the audit properly detects this​"
  - ​A result will be entered for every test, including the date completed and the initials of the tester
    - Results can be "PASS", "FAIL", "INCONCLUSIVE" or "FURTHER INVESTIGATION REQUIRED"
    - A note of "INCIDENTAL FINDING" can also be entered here for items that were not specifically part of the validation, but that require attention
  - ​Items will be color coded for ease of quick review: PASS, FAIL, INCONCLUSIVE, or FURTHER INVESTIGATION REQUIRED​, INCIDENTAL FINDING​
  - Any item that does not pass will be discussed as soon as is practical with the programming team
    - Collectively the teams may choose to pause implementation of the failing item, or the programming team may pursue a fix
    - Any fix requires additional testing and documentation, sequentially noted in the validation planning worksheet
  - ​Upon completion of all testing, the validation planning worksheet is renamed "Chartist V x.x.x.x validation" and will be maintained on the SharePoint
  - Upon ​completion of testing, the validation team will change the status of any "Chartist Suggestions or Improvements" list​​ item to either "Testing Complete - Passed" or "Testing Completes - Needs Attention"​
  - Upon successful roll out the status of any "Chartist Suggestions or Improvements" list​​ item to either "Implemented" or "Closed"
